# The Association of Army and Navy Medical Officers of the Confederate States

**Published:** 1901-11

**Authors:** 


### The Association of Army and Navy Medical Officers of the Confederate States.

Will meet in Dallas next June, during the reunion to be held there by the United Confederate Vetersn' Association. Dr. H. A. Moseley, of Dallas, chairman of the Committee of Arrangements, has announced the following list of committees:

1. Local Committee of Arrangements at Dallas: Dr. H. A. Moseley, assisted by Dr. S. H. Stout.

2. Finance Committee: Dr. A. M. Elmore, Dallas, assisted by Dr. F. E. Daniel, Austin. [Declined.—Ed.]

3. Relief and Emergency Hospital: Dr. A. R. Barry, Weatherford, assisted by Dr. J. E. Wilson, Dallas.

4. Sanitary: Dr. J. H. Florence, Dallas, assisted by Dr. Seay.

5. ——: Dr. J. H. Terrell, assisted by Dr. E. J. Reeves.

6. Program: Dr. S. H. Stout, assisted by W. M. Young.

7. Badges: Dr. E. Dunlap, assisted by Dr. K. W. Field.

8. Reception: Drs. B. G. Campbell and Beddo.

9. Invitations: Dr. J. C. J. King, Waco, and Dr. Fisher.

